# The interplay between human herpes simplex virus infection and the apoptosis and necroptosis cell death pathways

**DOI:** 10.1186/s12985-016-0528-0

**Published:** 2016-05-06

**Authors:** Xiaoliang Yu, Sudan He

**Affiliations:** Cyrus Tang Hematology Center and Collaborative Innovation Center of Hematology, Jiangsu Institute of Hematology, the First Affiliated Hospital, Soochow UniversitY, Suzhou, China; Jiangsu Key Laboratory of Preventive and Translational Medicine for Geriatric Diseases, Soochow University, Suzhou, China

**Keywords:** Herpes simplex virus, Host defense, Cell death, Apoptosis, Necroptosis

## Abstract

Human herpes simplex virus (HSV) is a ubiquitous human pathogen that establishes a lifelong latent infection and is associated with mucocutaneous lesions. In multicellular organisms, cell death is a crucial host defense mechanism that eliminates pathogen-infected cells. Apoptosis is a well-defined form of programmed cell death executed by a group of cysteine proteases, called caspases. Studies have shown that HSV has evolved strategies to counteract caspase activation and apoptosis by encoding anti-apoptotic viral proteins such as gD, gJ, Us3, LAT, and the ribonucleotide reductase large subunit (R1). Recently, necroptosis has been identified as a regulated form of necrosis that can be invoked in the absence of caspase activity. Receptor-interacting kinase 3 (RIP3 or RIPK3) has emerged as a central signaling molecule in necroptosis; it is activated via interaction with other RIP homotypic interaction motif (RHIM)-containing proteins such as RIP1 (or RIPK1). There is increasing evidence that HSV R1 manipulates necroptosis via the RHIM-dependent inactivation or activation ofRIP3 in a species-specific manner. This review summarizes the current understanding of the interplay between HSV infection and cell death pathways, with an emphasis on apoptosis and necroptosis.

## Background

Herpes simplex virus(HSV) is a ubiquitous human pathogen from the alpha-herpesvirinae subfamily [1]. There are two serotypes of HSV: HSV type 1 (HSV-1) and HSV type 2(HSV-2). It is a well-documented fact that the seropositivity rates for HSV-1 and HSV-2 in the general adult population are around 90 % and 25 %, respectively. HSV-1 is primarily associated with oral-labial lesions, whereas HSV-2 is the main cause of genital herpes [1, 2]. Rarely, severe infection of HSV-1 leads to fatal sporadic encephalitis [3]. HSV contains a large double-stranded DNA genome of around 150 K base pairs. There is around 83 % sequence homology of the protein-coding regions between HSV-1 and HSV-2 [4]. Therefore, HSV-1 and HSV-2 exhibit numerous biological similarities.

Cells have an innate capacity to activate effective antiviral countermeasures that can limit viral replication and viral dissemination. Among these antiviral responses, cell death is a common host defense mechanism against viral infection that eliminates virus-infected cells before the production of progeny virions. As would be expected, viruses tend to develop an ability to evade cell-death-based defenses; this evasion ability is generally viewed as beneficial to viral infection and pathogenesis [5]. It has been shown that HSV can establish a latent infection in the human peripheral nervous system for the entire life of the host. This infection can reactivate and trigger recurrent disease [6]. A growing body of evidence suggests that both HSV-1 and HSV-2 have evolved various strategies to manipulate host cell death signaling pathways, particularly the apoptosis and necroptosis pathways.

Apoptosis is a prevalent form of programmed cell death. Apoptosis has been shown to be vital for development and tissue homeostasis in multicellular organisms. The apoptotic cell displays characteristic morphological features including membrane blebbing, chromatin condensation, intra nucleosomal DNA fragmentation, and the formation of apoptotic bodies [7, 8]. Apoptosis is executed by a specific family of cysteine proteases, known as caspases [9]. HSV, like many other pathogenic viruses, encodes anti-apoptotic viral proteins including gD, gJ, Us3, latency-associated transcript (LAT) and ribonucleotide reductase large subunit (R1) to interfere with caspase activation [10, 11].

Necroptosis is morphologically characterized by membrane rupture and organelle swelling. It was traditionally thought to be an unregulated form of cell death caused by accidental physicochemical stresses. Recently, necroptosis has been identified as a regulated form of necrosis; this pathway can be activated in the absence of caspase activity [12]. Necroptosis is driven by the activation of receptor-interacting kinase 3(RIP3 or RIPK3) [13–15]. The RHIM domain of RIP3 is essential for its activation, acting to receive upstream signals through RHIM-dependent interactions [13, 14]. Interestingly, the viral M45-encoded RHIM-containing viral inhibitor of RIP activation protein (vIRA) in murine cytomegalovirus (MCMV), a herpesvirus from the beta-herpesvirinae subfamily, has been shown to prevent the activation of RIP3 [16, 17]. vIRA disrupts the RHIM-dependent interaction between RIP3 and the DNA-induced activator of interferon protein (DAI) [16, 17]. More recently, multiple studies have revealed that HSV R1 is capable of manipulating necroptosis signaling through RHIM-dependent modulation of RIP3 [18–21].

In this review, we summarize the current knowledge about the molecular mechanisms of apoptosis and necroptosis, and discuss how HSV manipulates these major cell death signaling pathways.

### Apoptosis signaling

**Fig. 1:**
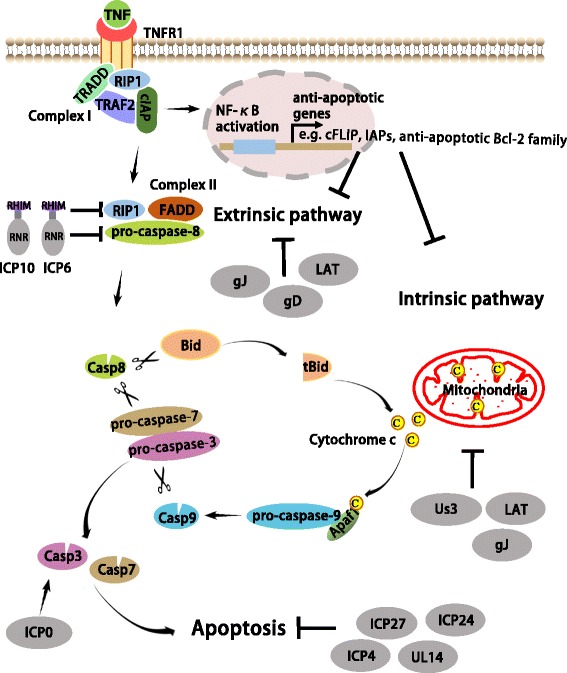
HSV modulates apoptosis signaling. Apoptosis is activated through both extrinsic and intrinsic pathways. In the extrinsic pathway, the ligation of a death receptor (e.g. tumor necrosis factor receptor-1; TNFR1) by its ligand promotes the assembly of the membrane-associated Complex I, which is composed of TRADD, TRAF2, RIP1, and cIAPs. This complex can activate NF-κB that initiates the transcription of some anti-apoptosis genes, such as cFLIP, IAPs and anti-apoptotic Bcl-2 family. During apoptosis, Complex I eventually triggers formation of Complex II, which is composed of FADD, RIP1, and pro-caspase-8, and leads to the activation of caspase-8. In the intrinsic pathway, cytochrome c is released from mitochondria to the cytoplasm, causing the formation of the apoptosome with Apaf-1 and pro-caspase-9, resulting in the activation of caspase-9. Activated caspase-8 and caspase-9 in turn cleave and activate downstream executioner caspases such as caspase-3 and caspase-7 for the execution of apoptosis. Activated caspase-8 also can engage the intrinsic pathway via cleavage of Bid to form tBid. HSV encodes anti-apoptotic viral proteins to subvert apoptotic signaling. Us3 can block the intrinsic pathway while R1 and LAT prevent the extrinsic pathway. gJ and LAT blocks both the intrinsic and the extrinsic pathways. gD activates NF-κB to enhancing the expression of anti-apoptotic genes. The regulatory proteins ICP4, ICP24 and ICP27 may indirectly inhibit apoptosis by promoting the production of later anti-apoptotic viral gene products. UL14 has HSP-like functions that may block caspases activation and apoptosis. Conversely, ICP0 promotes caspase activation and apoptosis

Apoptosis can be induced by a variety of stimuli through the activation of either cell surface death receptors (the ‘extrinsic pathway’) or mitochondrial effectors (the ‘intrinsic pathway’) (Fig. 1). The extrinsic apoptosis pathway is initiated through the binding of death ligands of the tumor necrosis factor (TNF) superfamily of cytokines, including TNFα, the TNF-related apoptosis-inducing ligand (TRAIL), and the CD95 (APO-1/Fas) ligand to their respective receptors, TNFR1, DR4/5, and Fas [22, 23]. As one example, the bingding of TNFα to TNFR1 promotes the assembly of a membrane signaling complex called Complex I that is composed of TNFR1-associated death domain protein (TRADD), TNFR-associated factor 2 (TRAF2), RIP1, and cellular inhibitors of apoptosis (cIAPs) [24]. Complex I functions to enable the nuclear translocation of the nuclear factor-κB (NF-κB), thereby activating the NF-κB signaling pathway. Activated NF-κB triggers the expression of anti-apoptotic genes, including genes encoding members of the cellular FLICE-inhibitory protein (cFILP) family [25], genes of the anti-apoptotic Bcl-2 family [26, 27], and genes of the inhibitor of apoptosis protein (IAP) family [28]. During apoptosis, Complex I eventually triggers formation of a signaling complex (termed Complex II) in the cytosol that is comprised of Fas-associated death domain (FADD), RIP1, and pro-caspase-8 [29]. This process results in the cleavage and activation of caspase-8 [29]. The activated caspase-8 in turn cleaves and activates downstream executioner caspases such as caspase-3 and caspase-7; or, alternatively, activated caspase-8 engages the intrinsic apoptosis pathway via cleavage of Bid to form tBid [30]. The extrinsic apoptosis pathway can be negatively regulated by IAPs and cFLIP. The IAP family proteins such as XIAP, cIAP1, and cIAP2 have been shown to bind caspases to block their activation [31]. cFLIP is highly homologous to pro-caspase-8 and thus has the ability to form a heterodimer with pro-caspase-8. This heterodimerization results in the inhibition of pro-caspase-8 activation [32].

In contrast, the intrinsic apoptosis pathway is initiated by intrinsic stimuli through mitochondrial signaling [33]. Upon apoptotic stimuli, mitochondrial outer-membrane permeabilization is induced. This triggers the release of cytochrome c from mitochondria to the cytoplasm. Subsequently, cytochrome c associates with apoptotic protease activating facter-1(Apaf-1) and pro-caspase-9 to form a protein complex termed the apoptosome. The formation of the apoptosome results in the activation of caspase-9 [34]. As with caspase-8, activated caspase-9 triggers a cascade of executioner caspases, leading to apoptosis [33]. The intrinsic apoptosis pathway is tightly regulated by the members of the Bcl-2 protein family, which can be divided into the anti-apoptotic Bcl-2 family members including Bcl-2, Bcl-xL, Mcl-1, and the pro-apoptotic Bcl-2 family members including Bid, Bax, and Bak [35].

### Modulation of apoptosis by HSV

Apoptosis is currently regarded as a critical cellular defense mechanism against viral invasion [10, 36]. Given that the elimination of infected cells via apoptosis limits viral replication and spread, it is not surprising that HSV has evolved apoptosis evasion strategies. Emerging evidence has established that HSV encodes anti-apoptotic viral proteins to subvert apoptosis (Fig. 1). The immediate-early genes including *ICP4*, *ICP27*, and *ICP24* are capable of inhibiting apoptosis during HSV infection. We know this because HSV recombinant viruses lacking each of these genes have an increased ability to initiate apoptosis compared to wild-type viruses [37–39]. Loss of either ICP4 or ICP27 also attenuates expression of early and late viral gene products, demonstrating that ICP4 and ICP27 are regulatory proteins in HSV [40, 41]. The N-terminal region of ICP27 is important for RNA binding and nuclear localization, and its C-terminal region is essential for the expression of early and late viral gene products. It has been demonstrated that the C-terminal region of ICP27, but not its N-terminal region, is required for the prevention of apoptosis during HSV infection [42]. Therefore, ICP27 likely has an indirect role in inhibiting apoptosis through enhancing the expression of early and late anti-apoptotic viral gene products.

Numerous studies have shown that early gene products, including Glycoprotein D (gD), Us3 and R1, are able to suppress apoptosis [43–46]. HSV strains that lacks gD exhibit a reduced ability to block apoptosis, while complementation with re-expression of gD restores the apoptotic phenotype of a gD-deficient virus [44]. One known gD receptors is herpes virus entry mediator (HVEM/TNFRSF14), which is a member of the TNF receptor family, and is capable of activating the NF-κB signaling pathway [47]. It has been shown that gD-mediated inhibition of Fas-induced apoptosis requires NF-κB activation to promote expression of anti-apoptotic genes [48]. Us3, a serine/threonine kinase, can affect intrinsic apoptosis signaling, as overexpression of Us3 inhibits cytochrome c release; this also inhibits caspase-3 activation in cells infected with ICP4-deficient HSV-1 [49]. Further Us3 has been shown to interact with programmed cell death protein 4 (PDCD4), and knockdown of PDCD4 can block apoptosis induced by ICP4-deficient HSV-1 [50]. In addition, some studies have shown that Us3 is able to phosphorylate the pro-apoptotic proteins Bad and Bid to block their function in promoting apoptosis [51–53]. The R1 proteins ICP6 and ICP10, have been well characterized as viral inhibitors of apoptosis. Both ICP6 and ICP10 contain an N-terminal RHIM-like domain [54] and a C-terminal ribonucleotide reductase (RNR) domain [55]. Despite having RNR activity, ICP6 is not required for HSV-1 growth or DNA replication in dividing cells [56]. Interestingly, cells infected with the HSV ICP6 deletion mutant were sensitive to poly(I:C)-induced apoptosis, which requires receptor-interacting protein 1 (RIP1) and TIR-domain-containing adapter-inducing interferon β (TRIF) [57]. The association of RIP1 and TRIF depends on the RHIM domains of both proteins. Of note, HSV R1 is able to block RHIM-dependent interaction between RIP1 and TRIF; it is also able to block apoptosis triggered by TRIF or RIP1 overexpression [57]. Moreover, expression of ICP6 or ICP10 provides protection against TNFα- and FasL-induced apoptosis [46, 58–61]. The RNR domain of HSV R1 can directly bind to the caspase-8 death effector domain and prevent caspase-8 activation, leading to suppression of extrinsic apoptosis signaling [46, 58–61]. It has been shown that ICP10 contains a serine–threonine protein kinase (PK) domain at its N-terminus [62]. The functional activity of PK is required for ICP10-mediated prevention of neuronal apoptosis, both incultured cells and in an in vivo model of N-methyl-D-aspartate (NMDA)-induced excitotoxicity [63, 64]. In the late phases of HSV replication, glycoprotein J is encoded by Us5. Deletion of gJ in both HSV-1 and HSV-2 leads to defects in inhibition of caspase activation in Fas-or UV-induced apoptosis [65]. Expression of gJ is able to inhibit Fas- or UV-induced activation of caspases-3, 6, 8, and 9 [65]. In addition to inhibiting Fas-mediated apoptosis, gJ can protect T lymphocytes against grB-mediated apoptosis [65]. The late gene UL14 product has been shown to inhibit apoptosis [66]. An HSV-1 UL14 protein deletion virus strain exerted decreased suppression of apoptosis compared to a rescued virus strain. UL14 has heat shock protein(HSP)-like functions that may play a role in apoptosis inhibition, as HSPs such as HSP70 and HSP27 are known to block caspase activation and apoptosis [67–69]. In addition, the latency-associated transcript (LAT) has been shown to protect neuronal cells against apoptosis, both in cultured cells and in in vivo animal models of HSV-1 latency [70–73]. Expression of LAT is able to inhibit caspase-8- and caspase-9-induced apoptosis, thereby interfering with both the extrinsic and the intrinsic apoptosis pathways [70, 73]. Such an inhibitory effect of LAT on apoptosis promotes neuronal survival in the latency-reactivation cycle and thus enhances spontaneous reactivation.

Although HSV has developed various strategies to interfere with apoptosis by encoding multiple anti-apoptotic viral proteins, the viral immediate-early ICP0 gene product has actually been identified as an apoptotic inducer during HSV-1 infection [74]. Wild-type HSV-1 infection is able to trigger apoptosis in the presence of the translational inhibitor cycloheximide (CHX), while the recombinant virus HSV-1(d109) that has deletions for all five α/IE genes fails to induce apoptosis [75, 76]. Deleting ICP0, but not ICP4 or ICP22, reduces the ability of HSV-1 to trigger apoptosis in the presence of CHX [74]. Moreover, combinant virus HSV-1 producing ICP0 is sufficient to trigger caspase-3 activation and apoptosis during viral infection [74]. It is conceivable that HSV-1 induces apoptosis, but then prevents the lethal effect of apoptosis on infected cells by producing anti-apoptotic viral proteins. These findings raise the possibility that HSV-1 may benefit from ICP0-mediated activation of apoptotic signaling during infection. However, this potentially beneficial function has not been fully elucidated.

### Necroptosis signaling

**Fig. 2:**
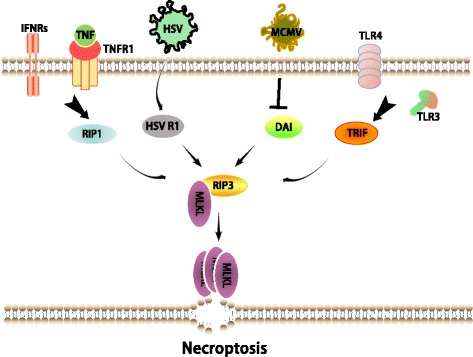
Necroptosis signaling pathways. Necroptosis can be induced through multiple signaling pathways that converge on RIP3. RIP3 is activated through interaction with a RHIM-containing protein in the following pathways: RIP1 (TNF family death receptors and type I IFNRs); TRIF (TLR3/4); DAI (M45/vIRA) mutant MCMV infection); HSV R1 (HSV infection in the mouse cells). Activated RIP3 phosphorylates its substrate MLKL and this event drives MLKL oligomerization and translocation to the plasma membrane, leading to necroptosis

Necroptosis can be activated by the TNF family death receptors, Toll-like receptors, and interferon receptors [77–79] (Fig. 2). The most extensively-studied necroptosis pathway is the one induced by the cytokine TNFα via the activation of TNF receptor 1(TNFR1). When TNFα binds to TNFR1, RIP1 is recruited to the TNFR1 complex (Complex I) and becomes ubiquitinated [77]. Cylindromatosis (CYLD) is a deubiquitylating enzyme that can remove the polyubiquitin chain from RIP1 [80]. Deubiquitination of RIP1 by CYLD triggers the disassociation of RIP1from TNFR1 and the subsequent formation of Complex II, which is comprised of FADD, RIP1, and caspase-8, leading to caspase-8 activation and apoptosis [29]. When caspase-8 activity is inhibited by chemical or viral inhibitors, TNF-induced apoptosis switches to necroptosis. In this process, RIP1 interacts with RIP3 to form a protein complex, termed a necrosome, through the RHIM domains of both proteins [13, 14]. Necrosome formation leads to the phosphorylation and activation of RIP3. Subsequently, the activated RIP3 phosphorylates its substrate MLKL [81, 82]. Upon phosphorylation, MLKL oligomerizes and translocates to the plasma membrane, eventually leading to necroptosis [83–85].

In addition, DAI and TRIF have been shown to activate RIP3 in the necroptosis pathways that are initiated, respectively, by M45/vIRA mutant MCMV and the activation of TLR3/4 [16, 78]. Notably, TRIF and DAI are able to mediate RIP3-dependent necroptosis independent of RIP1 [16, 78].

### Modulation of necroptosis by HSV

When caspase activity is impaired, necroptosis can serve as an alternate form of cell death to limit viral replication. Therefore, it will be interesting to see whether HSV has evolved strategies to subvert necroptosis for its sustainable replication. Intriguingly, recent studies have revealed that both HSV-1 and HSV-2 infection attenuated TNF-induced necroptosis in human cells. The expression of HSV-1 ICP6 or HSV-2 ICP10 is sufficient to disrupt TNF-induced necroptosis of human cells [20, 21]. The RHIM domains of ICP6 and ICP10 are required for their association with RIP1 or RIP3 [18]. Interaction between HSV R1 and RIP1 or RIP3 prevents the formation of the RIP1-RIP3 necrosome as well as the activation of RIP3 [20]. Notably, the RHIM domains of ICP6 and ICP10 are essential for their suppression capacity of RIP3 and necroptosis in human cells [20]. In addition to its role in inhibiting caspase-8-dependent apoptosis, the RNR domain of HSV R1 is required for the prevention of necroptosis in human cells [20]. Thus, HSV has an evolved a strategy to counteract the necroptosis response in human hosts through the HSV R1-mediated inactivation of RIP3.

**Fig. 3:**
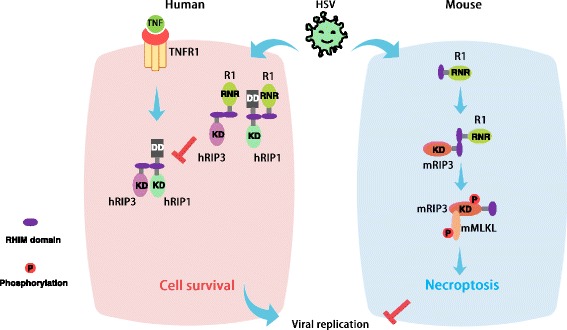
HSV modulates necroptosis signaling. HSV R1 can interact with RIP1 and RIP3 via their RHIM domains. In the natural human host, HSV R1 binding to RIP3 or/and RIP1 prevents the formation of the RIP1- RIP3 necrosome and also prevents the activation of RIP3. This effect counteracts necroptosis and promotes cell survival during HSV infection and thus facilitates efficient viral replication. In contrast, the association of R1 with RIP3, in the non-natural mouse host, drives the activation of RIP3 and necroptosis of infected cells, thereby limiting viral replication

Although HSV R1 exerts a RHIM competitor function to evade necroptosis in human cells [20, 21], both HSV-1and HSV-2 infection efficiently activate RIP3-dependent necroptosis in mouse cells [18, 19] (Fig. 3). This activated necroptosis pathway that follows HSV infection occurs independent of TNFR, TLR3, and DAI [18]. Strikingly, an ICP6 deletion mutant HSV-1 strain fails to trigger efficient necrotposis compared to the wild-type virus strain. Introduction of ICP6 into mouse cells is able to trigger RIP3-dependent necroptosis in a RHIM-dependent manner [18, 19]. These findings reveal an opposite impact of HSV R1 on necroptosis in mouse cells versus human cells. In mouse cells, induction of RIP3-dependent necroptosis leads to restriction of viral replication. Moreover, loss of RIP3 in mouse results in increased HSV-1 viral titers and mortality [18, 19]. Therefore, RIP3-dependent necroptosis acts as a crucial host defense mechanism to limit HSV replication in mouse, a non-natural host.

The species-specific modulation of necroptosis by HSV is consistent with the fact that humans are the natural host for HSV. In human cells, HSV R1 behaves like the RHIM-containing MCMV viral protein vIRA, which also has the ability to disrupt the RHIM-dependent activation of RIP3 [16]. Without vIRA, MCMV infection activates necroptosis through DAI-mediated activation of RIP3 in its natural mouse host. Thus, vIRA is required for MCMV replication by preventing the RHIM-dependent interaction between RIP3 and DAI in infected cells [16]. Additionally, some other herpes viral proteins, including the R45 and E45 proteins, contain a RHIM domain. It will be interesting to evaluate their impacts on necroptosis in natural and non-natural hosts. These studies will enhance our knowledge of pathogen-host interactions and deepen our understanding of the pathogenic mechanisms of infectious diseases.

## Conclusion

Apoptosis and necroptosis have been identified as critically-important host defense mechanisms contributing to the elimination of pathogen-infected cells. HSV has evolved various strategies to evade these cell death responses by encoding potent viral inhibitors. Among these, HSV R1 has been well-characterized as a suppressor of both caspase-8-induced apoptosis and RIP3-induced necroptosis in natural human hosts. It is noteworthy that necroptosis is activated in non-natural mouse host cells following HSV infection via the RIP3-mediated recognition of HSV R1. Therefore, it appears that HSV R1 modulates necroptosis via the RHIM-dependent activation or suppression of RIP3 signaling in a species-specific manner. Viral and cellular RHIM sequences seem to be determinants of whether a pro-necroptotic or an anti-necroptotic pathway is induced during HSV infection. Further understanding of the precise molecular mechanism(s) for this species-specific modulation of necroptosis by HSV will provide important insight into the development of novel therapeutic strategies for preventing the establishment of the latent HSV infection and viral spread.
